# ApoA-I/HDL Generation and Intracellular Cholesterol Transport through Cytosolic Lipid-Protein Particles in Astrocytes

**DOI:** 10.1155/2014/530720

**Published:** 2014-08-13

**Authors:** Jinichi Ito, Makoto Michikawa

**Affiliations:** Biochemistry, Graduate School of Medical Sciences, Nagoya City University, Kawasumi 1, Mizuho-cho, Mizuho-ku, Nagoya 467-8601, Japan

## Abstract

Exogenous apolipoprotein A-I (apoA-I) associates with ATP-binding cassette transporter A1 (ABCA1) on the cell surface of astrocytes like various peripheral cells and enhances the translocation of newly synthesized cholesterol from the endoplasmic reticulum/Golgi apparatus (ER/Golgi) to the cytosol. The cholesterol translocated to the cytosol is incorporated to cytosolic lipid-protein particles (CLPP) together with phospholipids and proteins such as sphingomyelin, phosphatidylcholine, caveolin-1, protein kinase C*α* (PK-C*α*), and cyclophilin A. The CLPP are high density lipoproteins- (HDL-)like cytosolic lipid-protein complex with densities of 1.09–1.16 g/mL and diameters of 17-18 nm. The association of exogenous apoA-I with cellular ABCA1 induces tyrosine phosphorylation, activation, and translocation to the CLPP of ABCA1-associated phospholipase C*γ* (PL-C*γ*) in rat astrocytes. Furthermore, PK-C*α* is translocated and activated to/in the CLPP through theproduction of diacylglyceride in the CLPP. ApoA-I enhances both the association of CLPP with microtubules and the phosphorylation of *α*-tubulin as a component of microtubules. The CLPP are dissociated from microtubules after *α*-tubulin in microtubules is phosphorylated by the CLPP-associated PK-C*α*. The association and dissociation between CLPP and microtubules may participate in the intracellular transport of cholesterol to the plasma membrane.

## 1. Introduction

Many lines of evidence of the relationship between cholesterol metabolism and diseases such as atherosclerosis [1–3], Tangier disease [4, 5], Niemann-Pick disease type C [6, 7], and Alzheimer's disease [8, 9] led to an erroneous notion that cholesterol is harmful to human health. Therefore, these lines of evidence indicate that cholesterol homeostasis is significant for the maintenance of human health. Cholesterol is an important lipid component of the cell membrane and regulates the fluidity and viscosity of the cell membrane by localizing among hydrocarbon chains of phospholipid molecules in the lipid bilayer [10, 11]. Cholesterol molecule, furthermore, constructs membrane lipid rafts along with sphingomyelin, which are cholesterol- and sphingomyelin-rich microdomains and are platforms for various proteins participating in signal transductions through the plasma membrane [12, 13].

Various types of cells generally synthesize cholesterol to keep membrane cholesterol level for the maintenance of cell function and survival. The cells also obtain cholesterol from low density lipoproteins (LDL) in the blood through the interaction with the LDL receptor on the cell surface [14]. The incorporated cholesterol molecule is hardly digested in almost all the cell types besides the cell types such as hepatocytes and steroid hormone-producing cells, because of a stubborn cyclopentanoperhydrophenanthrene structure of the cholesterol molecule. Therefore, excess cellular cholesterol and oxidative cholesterol must be allowed to extrude to the extracellular space and be transported to the liver [15]. Investigating the mechanisms underlying intracellular cholesterol transport and release of cholesterol to the extracellular space as high density lipoproteins (HDL) is very important for clarifying cholesterol homeostasis for the maintenance of cellular cholesterol level and prevention of cholesterol hyperaccumulation. However, the analysis of intracellular localization and movement of the cholesterol molecule after cholesterol biosynthesis is technically very difficult. The detailed mechanisms underlying the intracellular transport and efflux of de novo synthesized cholesterol through the interaction with exogenous apolipoproteins remain unclarified despite many previous investigations.

The central nervous system (CNS) is rich in cholesterol, which accounts for 20–25% of the total body cholesterol in humans, and neurons in the CNS are highly dependent on cholesterol for their survival and function. In cultured cell systems, suppression of cholesterol synthesis decreases the viability of neurons and addition of cholesterol as apoE/HDL results in resumption of synapse formation and neurite outgrowth [16–19], suggesting that apoE functions as an intercellular cholesterol transporter in the brain. However, the CNS in vertebrates is segregated by the blood-brain barrier (BBB) from the systemic circulation; therefore, apoE in CSF seems to be produced within the CNS [20]. Interestingly, recent studies have shown that apoE regulates BBB formation to control the integrity of tight junctions of BBB in an apoE-isoform-dependent manner* in vitro* and* in vivo* [21, 22]. Because BBB is a site for brain A*β* clearance to the blood, apoE-isoform-specific regulation of BBB function(s) may explain the mechanism underlying apoE4-accelerated development of Alzheimer disease.

Astrocytes in the CNS not only produce and secrete apolipoprotein E (apoE) as HDL containing apoE (apoE/HDL) [23, 24] but also generate apoA-I/HDL through the interaction of cell surface with exogenous apoA-I [25, 26]. As the most abundant apolipoproteins in human cerebrospinal fluid (CSF) are apoE and apoA-I [27–29], astrocytes are considered to play an important role in intercellular cholesterol transport in the CNS. We have been studying the mechanism underlying cholesterol efflux and intracellular cholesterol transport in astrocytes to clarify cholesterol homeostasis in the CNS. We found that HDL-like particles (cytosolic lipid-protein particles, CLPP) composed of lipid-protein complex in the cytosol participate in intracellular cholesterol transport through the interaction between CLPP and microtubules in astrocytes treated with exogenous apoA-I [30–32]. In this review, the mechanism underlying intracellular cholesterol transport in astrocytes treated with exogenous apolipoproteins, particularly apoA-I, is described on the basis of experimental data obtained in our laboratory.

## 2. Production and Function of ApoE and ApoA-I in the Brain

There are many studies of the mechanism underlying apoE secretion using macrophages [33–38]. After the biosynthesis in the ER, some of the apoE molecules are secreted from the plasma membrane through O-linked glycosylation in the Golgi [37], while other large parts of apoE are digested in the lysosomes. In macrophages, the secreted apoE is reinternalized by macrophages for the generation of apoE/HDL [34, 38]. ApoE appears to be secreted from macrophages to depend on the functions of protein kinase A and microtubules via a calcium-dependent pathway [36].

The mechanisms underlying apoE secretion and apoE/HDL generation in the brain are unclear at present. We have been studying the mechanism underlying apoE secretion using astrocytes prepared from the fetal rat brain. We found that the apoE secretion starts within 30 min after the initiation of apoE synthesis in rat astrocytes. An endogenous apoE is localized in the lipid rafts/caveolae domains in the manner dependent on the interaction with ABCA1 in wild type of astrocytes. The apoE is distributed in the nonraft fraction in the membrane fraction from ABCA1-KO astrocytes. Wild-type mouse astrocytes secrete apoE as apoE/HDL with densities of 1.08–1.12 g/mL, whereas the astrocytes prepared from ABCA1-KO mice secrete apoE as insufficiently lipidated HDL with higher density (*d* = 1.12–1.17 g/mL), suggesting that the lipidation of apoE depends on the function of ABCA1 in the membrane lipid rafts of astrocytes [39].

HDL-associating apolipoproteins found in cerebrospinal fluid (CSF) are predominantly apoE and apoA-I, in addition to minor apolipoproteins such as apolipoproteins A-IV, D, and J [40, 41]. As apoA-I with a molecular weight of 27 kDa is the second most abundant apolipoprotein next to apoE in the cerebrospinal fluid (CSF), apoA-I is also considered to contribute to cholesterol homeostasis in the brain along with apoE. The source of apoA-I is not clear, while apoE and apoJ are produced and secreted by astrocytes. Although apoA-I mRNA is hardly found in the brain, there are a few reports that apoA-I is produced by the capillary endothelial cells in the brain [42, 43]. ApoA-I in the CNS may be derived also from plasma through the blood-brain barrier (BBB). Astrocytes do not apparently secrete apoA-I. It is possible for astrocytes to interact with exogenous apoA-I secreted by endothelial cells in the brain. We showed that rat astrocytes generate biochemically different HDL through exogenous apoA-I- and endogenous apoE-mediated cholesterol release, suggesting different roles between apoA-I/HDL and apoE/HDL in the brain. ApoA-I especially seemingly participates in removal of excess cholesterol from the brain to outside of the BBB to convert 24(S)OH-cholesterol as a more polar molecule [44]. As 24(S)OH-cholesterol is a high affinity ligand for LXR*α* and LXR*β*, this sterol enhances expression of ABCA1 to upregulate cholesterol efflux. Scavenger receptor class B type 1 (SR-B1) in endothelial cells effectively supports the reverse of apoA-I-mediated sterol transport. Such a system to transport cholesterol to outside of the brain through the BBB may be favorable for removal of excess cholesterol in the brain, as brain capillary endothelial cells form the BBB along with astrocytes.

The levels of cholesterol and apoA-I in plasma HDL have been known to be low in patients with Alzheimer's disease, suggesting that high levels of apoA-I/HDL reduce the risk of cardiovascular disease. Lewis et al. showed that overexpression of human apoA-I is effective to preserve cognitive function in a mouse model of Alzheimer's disease through reducing A*β*-induced production of chemokines/cytokines [45]. However, there are some reports that high levels of apoA-I/HDL in plasma are not effective to protect Alzheimer's disease [46]. Haploinsufficiency of angiogenin, furthermore, is a risk factor of Alzheimer's disease. Amyloidogenic variant of apoA-I apparently induces cell death through reducing angiogenin expression to attenuate antistress activity [47]. These findings suggest that apoA-I not only has a role to regulate cholesterol homeostasis in the brain but also protects the brain from injury and stress.

## 3. Generation of ApoA-I-Containing HDL in Astrocytes

Astrocytes synthesize and secrete apoE as cholesterol-rich apoE/HDL [24], which are taken up by neurons through apoE receptors. Although neurons produce cholesterol, additional amounts of cholesterol from astrocytes as apoE/HDL are required for the formation of numerous synapses in the CNS [18]. It has been reported that the production of apoE/HDL in the CNS also depends on ABCA1 [48–51]. However, it is unclear how ABCA1 regulates the secretion and lipidation of apoE in CNS cells.

On the other hand, astrocytes generate HDL through the interaction between exogenous lipid-free apoA-I and ABCA1 on their cell surfaces similarly to peripheral cells [52]. ApoA-I/HDL generated in astrocytes through the interaction with exogenous apoA-I is more cholesterol-poor and phospholipid-rich than apoE/HDL generated by endogenous apoE in astrocytes [25, 30]. The differences in features between apoA-I/HDL and apoE/HDL are considered to depend on differences in features between apoA-I and apoE. The mechanism underlying apoA-I/HDL generation may be different from that underlying apoE/HDL generation. Because apoA-I is an activator of lecithin-cholesterol acyltransferase (LCAT), which is expressed in the brain [53], apoA-I must participate in intercellular cholesterol transport in the CNS as in the case of peripheral cholesterol transport.

ApoA-I in the plasma enhances efflux of cholesterol and phospholipids from the cell surface of many kinds of peripheral cells to generate HDL through the interaction with ABCA1. ABCA1 is a membrane-penetrating protein with a molecular weight of 250 kDa [54–56], and its deficiency is known to cause Tangier disease without HDL in the plasma. There are two hypotheses for the mechanism underlying apoA-I-mediated cholesterol efflux through the interaction between exogenous apoA-I and ABCA1. One hypothesis is that apoA-I generates lipoproteins utilizing lipids such as cholesterol and phospholipids at the cell surface dependently on ABCA1 [57–60]. Landry et al. reported that ABCA1 expression results in a significant redistribution of cholesterol and sphingomyelin from rafts to nonrafts [61]. ABCA1 seems to form an unstable domain in the lipid structure of the plasma membrane to facilitate the removal of cholesterol from the cell surface. The other hypothesis is that apoA-I is internalized and then forms apoA-I-lipid complexes in late endosomes after the binding of apoA-I and ABCA1 at the cell surface [62, 63]. The mechanism underlying apoA-I-induced cholesterol efflux through the interaction between apoA-I and ABCA1, however, has not been clarified to date. It has been shown that lipid-free apoA-I generates pre*β*-HDL through the interaction with ABCA1 and that pre*β*-HDL matures to cholesterol-rich HDL with a lower density through their interaction with ABCG1 [64–66]. Karten et al. proposed that ABCG1 plays a role in cholesterol efflux mediated by exogenous apoA-I in astrocytes [67]. Taking all these lines of evidence together, the generation of apoA-I-mediated HDL may require the collaboration of ABCA1 and ABCG1 in astrocytes.

## 4. Exogenous ApoA-I-Mediated Intracellular Cholesterol Transport

We observed that the newly synthesized cholesterol and phospholipids such as phosphatidylcholine and sphingomyelin are transiently translocated from the ER/Golgi to the cytosol in rat astrocytes treated with exogenous human apoA-I for 90 min [31]. The cholesterol and phospholipids translocated to the cytosol were recovered with the cytosolic fraction with densities of 1.09–1.16 g/mL like plasma HDL [31]. Proteins such as caveolin-1 and cyclophilin A are also translocated to this fraction in the cytosol of rat astrocytes after the apoA-I treatment for 90 min. A cyclophilin A-specific inhibitor, cyclosporin A, suppressed not only the translocation of newly synthesized lipid and caveolin-1 to the cytosol but also the cholesterol efflux in apoA-I-treated rat astrocytes [31, 68]. These findings suggest that the newly synthesized cholesterol and phospholipids exist as lipoprotein-like particles together with proteins such as caveolin-1 and cyclophilin A in the cytosol. The electron microscopic observation showed HDL-like lipid-protein particles with diameters of 17-18 nm and we referred to them as cytosolic lipid-protein particles (CLPP) [31]. Because CLPP are soluble in Triton X-100, it is considered that CLPP are not derived from the lipid rafts/caveolae domain, although caveolin-1 is transported to the CLPP from the lipid rafts/caveolae domain.

**Figure 1:**
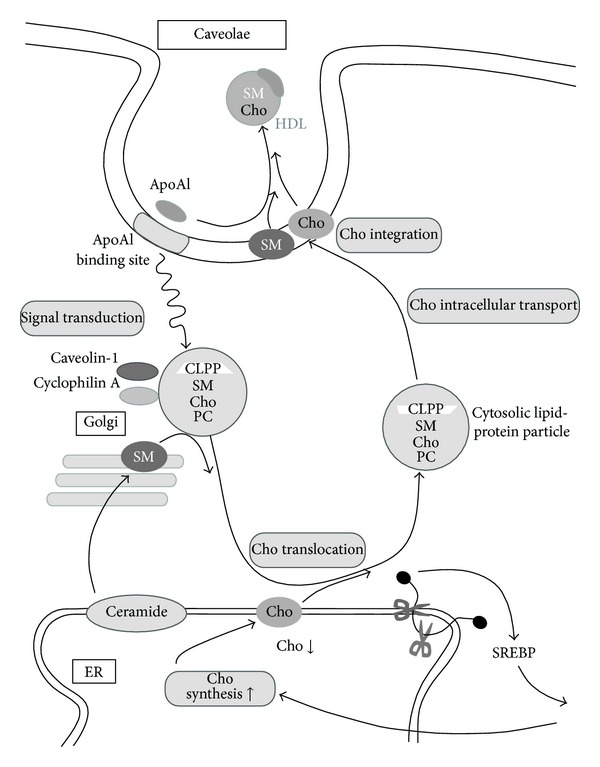
Putative mechanism underlying intracellular cholesterol transport in astrocytes. Cho: cholesterol, SM: sphingomyelin, PC: phosphatidylcholine, and apoA-I: apolipoprotein A-I.

As the syntheses of cholesterol and phospholipids are enhanced in rat astrocytes at 2 h after apoA-I treatment, it is suggested that the apoA-I-induced lipid translocation from ER/Golgi to cytosol precedes the increase of lipid synthesis. The processing of SREBP1 and SREBP2 more than 60 min after the treatment with apoA-I is actually enhanced and the mature forms of these proteins were transported to the nuclei [31]. The maturation of SREBPs may proceed to stimulate the transcription of enzymes relating to the syntheses of lipids such as cholesterol and phospholipids, because the cholesterol level in the ER membrane must be decreased by cholesterol translocation to the CLPP in the cytosol after the apoA-I treatment. These findings suggest that the collaboration between cyclophilin A and caveolin-1 is required in the intracellular transport of newly synthesized cholesterol from the ER/Golgi to the plasma membrane and apoA-I-mediated cholesterol efflux. The CLPP appear to function as vehicles for the intracellular transport of cholesterol (Figure 1).

We examined whether exogenous apoA-I associates with ABCA1 in rat astrocytes like peripheral cells. We found by western blotting analysis that apoA-I comigrated with ABCA1 with an apparent molecular weight of over 260 kDa in rat astrocytes treated with apoA-I and then with a crosslinker, BS3 [52]. ApoA-I-bound ABCA1 was isolated by anti-apoA-I antibody-bound Protein G/Sepharose.

The solubilized ABCA1 in the membrane fraction of rat astrocytes, furthermore, bound to apoA-I-immobilized AffiGel 15. LXR agonist, To901317, enhances both cellular level of ABCA1 and binding of apoA-I to ABCA1. Phospholipase C*γ* (PL-C*γ*) is coisolated with ABCA1 from the solubilized membrane fraction of rat astrocytes using apoA-I-immobilized AffiGel 15 or using anti-ABCA1 antibody-bound Protein G/Sepharose [52]. These findings suggest that exogenous apoA-I binds to the extracellular domain of ABCA1 that associates with PL-C*γ* at the intracellular site in astrocytes.

ApoA-I induces the tyrosine phosphorylation of PL-C*γ* in rat astrocytes at 5 min after apoA-I stimulation, followed by the translocation of PL-C*γ* to the CLPP fraction and diacylglyceride production in the CLPP. The SiRNA of ABCA1 suppressed not only the PL-C*γ* binding to ABCA1 but also the apoA-I-induced tyrosine phosphorylation of PL-C*γ*. The protein kinase C*α* (PK-C*α*) translocation to and its activation in the CLPP are also continuously enhanced in apoA-I-treated rat astrocytes [30]. The suppression of either phospholipase C or protein kinase C inhibits apoA-I-mediated cholesterol release and apoA-I/HDL generation. These findings suggest that the CLPP are intracellular sites of apoA-I-induced signal transduction and function as intracellular cholesterol transport vehicles.

## 5. Function of Sphingomyelin in Intracellular Cholesterol Transport

Many reports show that cholesterol is specifically associated with sphingomyelin rather than other phospholipids in the plasma membrane [69–71]. The membrane lipid rafts are cholesterol- and sphingomyelin-rich membrane microdomains and indicate an important feature that may contribute to signalings through the plasma membrane. Caveolin-1, furthermore, forms an invagination in cholesterol- and sphingomyelin-rich membrane microdomains as caveolae. It is expected that when cells are treated with sphingomyelinase (SMase), sphingomyelin is digested in the cell surface, which is followed by cholesterol redistribution and destruction of lipid raft/caveolae in the plasma membrane. Therefore, the treatment of astrocytes with SMase may influence apoA-I-mediated cholesterol release and intracellular cholesterol transport through the decrease in sphingomyelin level and the unstability of cholesterol localization in the plasma membrane. ApoA-I-mediated cholesterol efflux from the cell surface is actually enhanced in SMase-treated rat astrocytes. The addition of exogenous sphingomyelin suppressed conversely the apoA-I-mediated cholesterol release. De novo synthesis of sphingomyelin is enhanced after the SMase treatment and then apoA-I-mediated cholesterol efflux is gradually suppressed [72].

Sphingomyelin may function as an anchor of cholesterol in the plasma membrane, particularly in the lipid rafts/caveolae domain. This is also supported by the report by Nagao et al. that exogenously added sphingomyelin significantly inhibits the apoA-I-dependent cholesterol efflux from sphingomyelin-deficient Chinese hamster ovary cells [73].

Surprisingly, the suppression of sphingomyelin synthesis using D609 (a phosphatidylcholine-specific phospholipase C inhibitor) after the SMase treatment markedly inhibits apoA-I-mediated cholesterol release, which is contrary to our expectation. We examined how sphingomyelin participates in intracellular cholesterol transport in rat astrocytes. Our experimental results suggest that sphingomyelin is synthesized through the transfer of a phosphorylcholine group to ceramide from phosphatidylcholine in apoA-I-stimulated rat astrocytes like SMase-treated cells [32]. D609 suppressed not only apoA-I-mediated cholesterol efflux but also cholesterol translocation from the ER/Golgi to the cytosol without inhibiting HMG-CoA reductase activity [32]. This finding suggests that sphingomyelin functions as one of the factors that stimulate the translocation of newly synthesized cholesterol to the cytosol from the ER/Golgi for intracellular cholesterol transport to the plasma membrane in addition to an anchor of cholesterol to regulate apoA-I-mediated cholesterol efflux. It is considered that diacylglyceride, which is produced during sphingomyelin synthesis, participates in intracellular cholesterol transport through the activation of PK-C. We demonstrated on the base of other experiments that ApoA-induced PK-C*α* activation has a great role in the intracellular cholesterol transport in astrocytes.

## 6. Interaction between CLPP and Microtubules to Regulate Intracellular Cholesterol Transport and ApoA-I-Mediated Cholesterol Efflux

**Figure 2:**
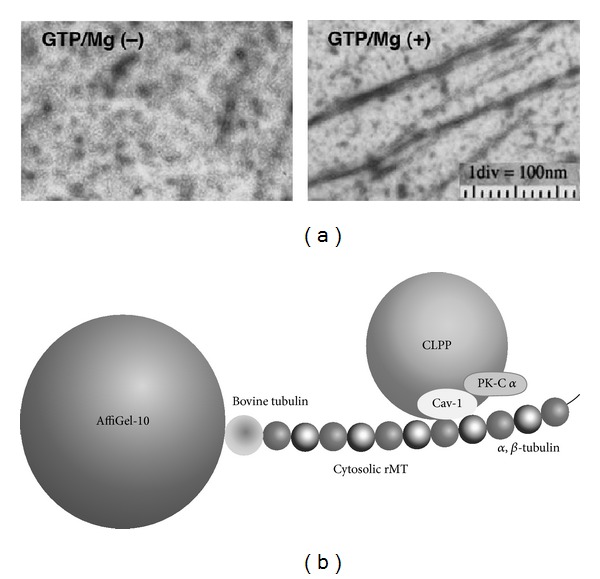
Reconstitution of microtubule-like filaments (rMT) using cytosol fraction prepared from rat astrocytes (a) and scheme of association of cytosolic lipid-protein particles (CLPP) and rMT through AffiGel-immobilized bovine tubulins.

We found that the interaction between CLPP and microtubules is important to promote intracellular cholesterol transport for apoA-mediated cholesterol efflux from rat astrocytes. The association of CLPP with microtubule-like filaments (rMT) reconstituted* in vitro* using the cytosol fraction prepared from rat astrocytes stimulated with apoA-I was examined in order to show the association of CLPP and microtubules. The cytosol fraction was incubated with bovine tubulin-immobilized AffiGel 10 (bovine tubulin/AffiGel) under the microtubule-polymerization condition to construct bovine tubulin/AffiGel-associated rMT. The binding of CLPP-related cytosolic caveolin-1 and PK-C*α* to bovine tubulin/AffiGel-associated rMT was more actively increased in the cytosol of rat astrocytes treated with apoA-I for 5 min than in the control cytosol without apoA-I treatment [74]. This finding suggests that apoA-I enhances the association of CLPP and microtubules in rat astrocytes (Figure 2). This association between rMT and CLPP is inhibited by addition of a scaffolding domain peptide of caveolin-1, suggesting that apoA-I enhances the association of microtubules and CLPP through the CLPP-binding caveolin-1. Furthermore, dysfunction of microtubules induced by Taxol suppresses not only the translocation of newly synthesized lipids such as cholesterol and phosphatidylcholine to the cytosol but also lipid release mediated by apoA-I. It is considered on the base of these findings that the intracellular cholesterol transport is promoted by microtubules in rat astrocytes treated with apoA-I. We thus suggested that intracellular cholesterol transport is mediated by cytosolic particles, CLPP, and regulated by the interaction between CLPP and microtubules in astrocytes, although there is still a possibility that cytosolic vesicles are involved in intracellular cholesterol transport.

**Figure 3:**
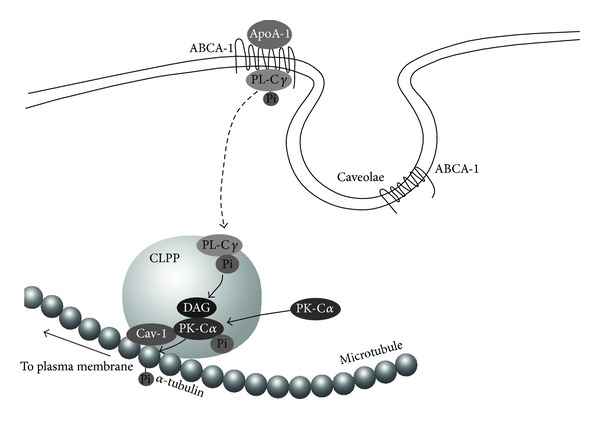
Hypothesis of intracellular transport of CLPP through phosphorylation of *α*-tubulin in microtubules after interaction between apoA-I and ABCA1.

We found that apoA-I enhances the phosphorylation of *α*-tubulin, which is suppressed by a protein kinase C inhibitor, bisindolylmaleimide 1 (BIM) [75]. BIM suppresses not only cholesterol translocation to the cytosol from the ER/Golgi but also apoA-I-mediated cholesterol efflux, implying that PK-C participates in intracellular cholesterol transport and cholesterol efflux through the phosphorylation of *α*-tubulin. BIM also suppressed the phosphorylation of the cytosolic caveolin-1-associated *α*-tubulin in apoA-I-stimulated astrocytes, suggesting that the caveolin-1-associated *α*-tubulin is phosphorylated by cytosolic caveolin-1-associated PK-C*α* in the CLPP. The phosphorylation of the *α*-tubulin induces the dissociation of *α*-tubulin from caveolin-1, and the phosphorylated *α*-tubulin never associates with caveolin-1. These findings suggest that apoA-I enhances the association of microtubules with CLPP, followed by the phosphorylation of *α*-tubulin and the dissociation of CLPP from microtubules. The *α*-tubulin phosphorylation induced by PK-C*α* may regulate the interaction between CLPP and microtubules for the intracellular cholesterol transport to the plasma membrane, cholesterol release, and apoA-I/HDL generation. The dephosphorylation of *α*-tubulin enhances this association, suggesting that the phosphorylation/dephosphorylation of the *α*-tubulin regulates intracellular cholesterol transport to the plasma membrane (Figure 3).

## 7. Conclusions

Cholesterol is a very important membrane lipid to keep function and survival of neurons in the brain. Astrocytes supply neurons with cholesterol through HDL generated by the interaction with endogenous apoE synthesized in astrocytes and with exogenous apoA-I derived from the brain capillary endothelial cells. We described the mechanisms for HDL generation and intracellular cholesterol transport in rat astrocytes stimulated with exogenous apoA-I in this paper.

We showed a hypothesis of the mechanism for intracellular transport of newly synthesized cholesterol to the plasma membrane from the ER/Golgi through cytosolic lipid-protein particles (CLPP) after the interaction between exogenous apoA-I and ABCA1 on the cell surface of rat astrocytes. CLPP are mainly composed of lipids such as cholesterol, phosphatidylcholine, and sphingomyelin and proteins such as caveolin-1, cyclophilin A, and protein kinase C*α*. After the interaction between apoA-I and ABCA1 in astrocytes, cholesterol and phospholipids biosynthesized in the ER/Golgi and caveolin-1 are translocated to the CLPP. Furthermore, the ABCA1-associated phospholipase C*γ* is activated and translocated to the CLPP. The CLPP are associated with microtubules through the CLPP-associated caveolin-1. The activated phospholipase C*γ* produces diacylglyceride in the CLPP, followed by translocation and activation of protein kinase C*α* to/in the CLPP. The activated protein kinase C*α* in the CLPP phosphorylates *α*-tubulin in the microtubules and then the CLPP go away from the phosphorylated *α*-tubulin in the microtubules. Thus, the CLPP are considered to be associated with next nonphosphorylated *α*-tubulin in microtubules to move to plasma membrane for the supply of newly synthesized lipids to the plasma membrane. Although our hypothesis shows intracellular cholesterol transport through particles of lipids-proteins complex but not vesicles, this does not deny other mechanisms for intracellular cholesterol transport through cellular vesicles.
